# Life after Stroke in an Urban Minority Population: A Photovoice Project

**DOI:** 10.3390/ijerph14030293

**Published:** 2017-03-11

**Authors:** Revathi Balakrishnan, Benjamin Kaplan, Rennie Negron, Kezhen Fei, Judith Z. Goldfinger, Carol R. Horowitz

**Affiliations:** 1 Division of Cardiology, New York University School of Medicine, 462 1st Avenue NBV 17S5, New York, NY 10016, USA; 2 University of North Carolina School of Medicine, 321 S Columbia St, Chapel Hill, NC 27516, USA; benjamin_Kaplan@med.unc.edu; 3 Yale Institute for Network Science, Department of Sociology, Yale University, 17 Hillhouse Avenue, New Haven, CT 06520, USA; rennie.negron@yale.edu; 4 Department of Population Health Science and Policy, Icahn School of Medicine at Mount Sinai, 1468 Madison Ave, New York, NY 10029, USA; kezhen.fei@mountsinai.org; 5 Zena and Michael A. Wiener Cardiovascular Institute, Icahn School of Medicine at Mount Sinai, One Gustave L. Levy Place, New York, NY 10029, USA; judith.goldfinger@mountsinai.org; 6 Center of Health Equity and Community Engaged Research at Mount Sinai, Department of Population Health Science and Policy, Icahn School at Mount Sinai, One Gustave L. Levy Place, New York, NY 10029, USA

**Keywords:** photovoice, qualitative research, stroke, Black/African-American, Hispanic

## Abstract

Stroke is a leading cause of disability in the United States and disproportionately affects minority populations. We sought to explore the quality of life in urban, minority stroke survivors through their own photos and narratives. Using the Photovoice method, seventeen stroke survivors were instructed to take pictures reflecting their experience living with and recovering from stroke. Key photographs were discussed in detail; participants brainstormed ways to improve their lives and presented their work in clinical and community sites. Group discussions were recorded, transcribed, and coded transcripts were reviewed with written narratives to identify themes. Participants conveyed recovery from stroke in three stages: learning to navigate the initial physical and emotional impact of the stroke; coping with newfound physical and emotional barriers; and long-term adaptation to physical impairment and/or chronic disease. Participants navigated this stage-based model to varying degrees of success and identified barriers and facilitators to this process. Barriers included limited access for disabled and limited healthy food choices unique to the urban setting; facilitators included presence of social support and community engagement. Using Photovoice, diverse stroke survivors were able to identify common challenges in adapting to life after stroke and important factors for recovery of quality of life.

## 1. Introduction

Stroke is one of the leading causes of disability in the United States and can result in a wide spectrum of effects on both physical and mental health. Recovery can be a long and difficult process for stroke survivors to navigate. A 2010 study of Northern Manhattan stroke survivors showed that functional independence was lost over a period of five years, independent of recurrent stroke and other risk factors [1]. Stroke survivors are also more likely to internalize and marginalize themselves socially from others [2].

The risk of recurrent stroke disproportionately affects minority populations, with African-Americans twice as likely and Hispanics one and a half times as likely to experience recurrent stroke compared to Caucasians [3]. In addition, there is limited insight into the full degree of impact this disease has in minority populations and the barriers they may face in recovery.

Photovoice is a qualitative research method that was developed to learn about personal experiences through the viewpoint of the study participant. Subjects are given cameras and are asked to present their own experience through photographic expression and narratives, guided by targeted questions [4]. This technique places data collection directly in the hands of the subject and allows them to capture, present, and narrate complex emotional and physical obstacles that may be otherwise difficult to verbalize. The results can then be used to highlight needs, inform, and influence policy to help effect social change.

In the past, the Photovoice technique has been used to study the experiences of marginalized communities, including homeless single mothers in Detroit and HIV-positive youth in Africa [5,6]. In these studies, Photovoice provided its participants with the opportunity to display their own day-to-day experiences and reach an audience to which they may have been otherwise isolated. Using Photovoice, we aimed to develop a greater understanding of recovery from stroke through photos and narratives grounded in the perspective of the stroke survivor. Specifically, we aimed to examine quality of life of urban, minority stroke survivors and identify barriers and facilitators that affect emotional and physical recovery.

## 2. Materials and Methods

### 2.1. Participants

Our team recruited participants from a cohort enrolled in a randomized controlled secondary stroke prevention trial: Prevent Return of All Inner City Strokes through Education (PRAISE) [7,8]. The inclusion criteria for the PRAISE trial were the following: age 40 and older and history of stroke or transient ischemic attack (TIA) within the past five years. Subjects were also required to be able to physically and cognitively participate in educational group sessions. For participation in Photovoice, patients were required to be able to operate a digital camera.

Subjects already enrolled in the PRAISE trial were approached for recruitment in the Photovoice study after the initial baseline enrollment survey, or as they returned for follow-up interviews and data collection at 6-month and 12-month visits for the larger trial. We enrolled 17 study participants in four different Photovoice groups. Each group participated in three separate sessions held between November 2010 and July 2011. Of these, we provided 15 with digital cameras and two chose to use their own cameras. We transcribed written notes and recorded audio during each meeting. We allowed participants to keep the cameras as a gift, and presented those who used personal cameras with a gift card equivalent to the camera value ($50). The Mount Sinai Medical Center Institutional Review Board (IRB) approved the study (GCO# 02-0515 0001 03). 

### 2.2. Photovoice Sessions

Trained moderators held one session to introduce the Photovoice method, provide brief photography training, and asked participants to use photographs to answer a question: What has made it easier or harder to live your daily life, be part of your community, and prevent having another stroke? During the second session, participants presented their top five photos to the group using the SHOWeD method to explore their personal experiences: (1) What do you See here? (2) What is Happening here? (3) How does this relate to Our lives? (4) Why does this situation or concern exist? (5) What can we Do about it? [9] During the third session, participants presented their photos and shared narratives with the group.

At the completion of the PRAISE trial in 2013, we administered follow-up phone calls to the Photovoice participants in order to obtain feedback focused on their experience within the Photovoice study; all were contacted with the exception of one who was deceased at the time of follow up.

### 2.3. Data Analysis

Using a grounded theory approach and content analysis method [10], data were reviewed and analyzed after each session and again after all sessions were complete. The content analysis method involved initial identification of meaningful text, then more in depth interpretation through coding, and categorization and identification of themes and subthemes [11]. Audio recordings from the second and third Photovoice sessions were transcribed and combined with notes from the sessions.

We utilized iterative review of the transcripts, session notes, written text, and coder triangulation in order to thematically analyze the data. We created a master list of themes and codes that included deductive themes developed from the literature review and the interactive group sessions and inductive themes from the session notes, transcripts, and text accompanying the photographs [12]. Relevant themes were identified by grouping similar codes. Manual methods for analysis were used. Two authors (who acted as group session facilitators) independently coded transcripts using the master list and a third author (who was not present during group sessions) reviewed the coded transcripts and found an inter-rater agreement of 76%. The three authors then discussed and resolved any disagreements through consensus. Member checking with participants was conducted at the end of the third group session to validate themes that were deduced between sessions two and three. The study was IRB approved and we de-identified all documents (removing participant names and replacing them with numbers) and kept all files in password-protected folders protected by a firewall.

## 3. Results

### 3.1. Demographics

Overall demographics of the participants can be seen in Table 1. The mean age was 64 years (±9.8) and mean time from stroke was 2.0 years (±1.5). Participants were mostly female (65%), and Black (65%). Utilizing modified Rankin scores collected from the PRAISE trial, it was found that 41% had moderate to moderately severe post-stroke disability; the remainder had slight or no disability. The score ranges from 0 to 6, where 0 is no disability and 6 is dead, and 5 is severe disability requiring continuous care [13].

### 3.2. Conceptual Model of Recovery

**Figure 1:**
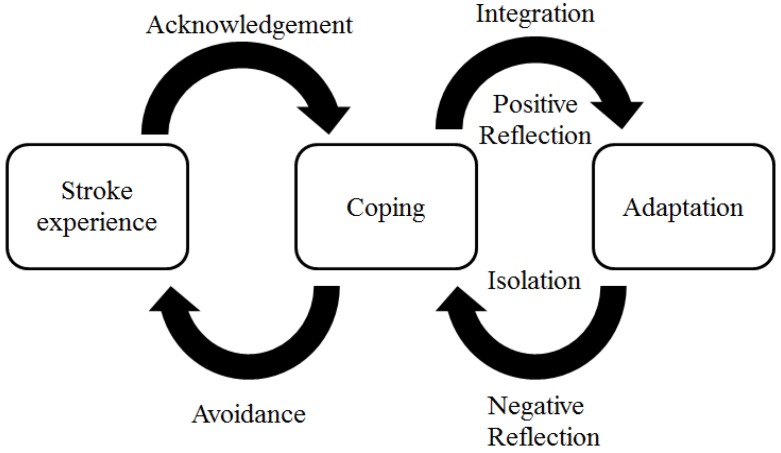
Conceptual model.

The photos and accompanying narratives varied in content but shared a common theme: a journey of recovery and adaptation to life after stroke. We present this major theme through a three-stage conceptual framework composed of the subthemes that emerged during the Photovoice sessions. As outlined in Figure 1, these stages include: (1) The initial stroke experience and reaction to the immediate effects of the stroke (acknowledgement versus avoidance); (2) coping strategies (integration versus isolation); and (3) long-term adaptation. Transitioning between the first and second stages was facilitated by acknowledgement of new challenges and hindered by avoidance of these same challenges. Transitioning between the second and third stages was facilitated by positive reflection on the personal experience and increased social integration, and was hindered by negative reflection and social isolation. Here, we explain each stage and transition of this framework as described by the participants, as well as the sub-themes that characterize each.

#### 3.2.1. Stroke Experience

During the group discussions (second session), nearly all participants shared the immediate emotional and physical impact of the stroke experience, focusing on the “How does this relate to our lives?” question of the SHOWeD method. Many felt a strong sense of shame and disappointment upon realizing the impact of their new disability. One younger woman described the initial impact, describing a photo of the entrance to her building:

“The day I came home it took me almost half an hour to get to the front door to the lobby. I was crushed. I wanted to just cry, crawl under a rock or just be somewhere there was no one... It wasn’t until then that I realized my life was over…”

#### 3.2.2. Coping: Acknowledgement versus Avoidance

After the initial stroke, most participants described a process of acknowledging the new emotional and physical barriers. Those able to cope with these barriers did so by either overcoming or accepting their newfound limitations. Others described a process of avoiding situations that would reveal their limitations to themselves and others, which was associated with sustained feelings of frustration.

• Environmental barriers

As all participants lived in densely populated urban neighborhoods, many of their photos depicted local environmental features that made living with a physical disability difficult. During the group discussions, participants identified these barriers through photographs, highlighting a lack of public benches which discouraged walking (no place to sit and rest) and a lack of subway elevators, which limited transportation options. Local disrepair, such as cracks in sidewalks, further discouraged outdoor exercise, posing threats to physical safety. Some participants found these obstacles isolating and avoided going outside, while others engaged them as motivational tools. One emphasized,

“This is an obstacle course... I found out you have to be twice as cautious and more tentative walking…and I didn’t want people to help me, I was sweatin’ bullets, but I had to take that challenge.”

The discussion and identification of environmental barriers revealed the common struggles that those who were physically disabled faced in navigating the urban environment, and fostered dialogue among the group to answer the “What can We Do about it?” question of the SHOWeD method.

• Emotional barriers

**Figure 2:**
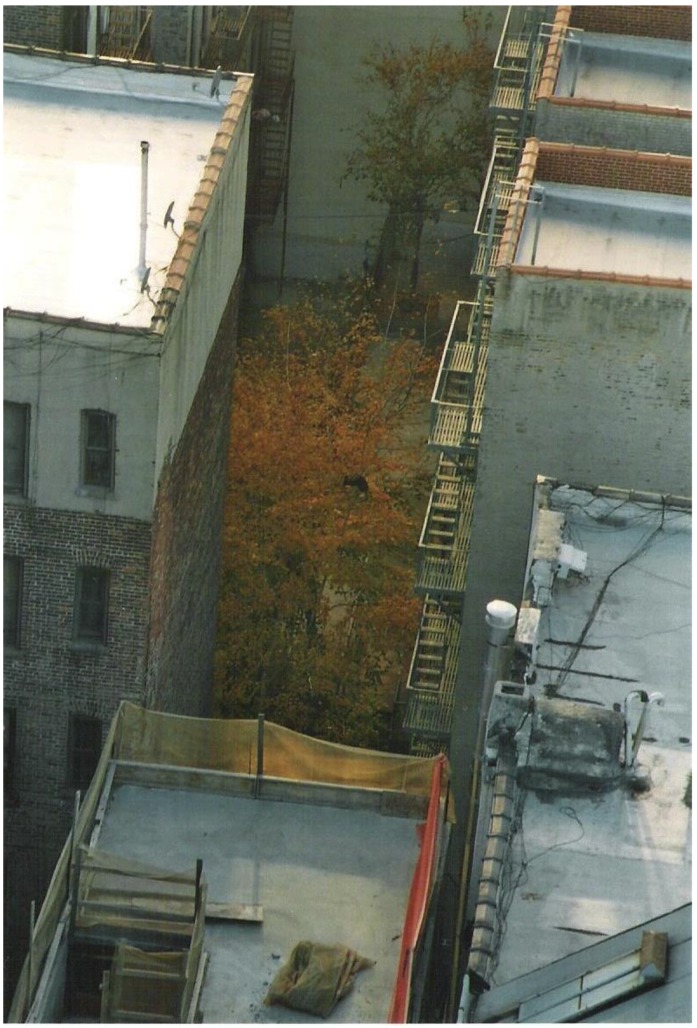
The tree.

Nearly all participants expressed difficulty in coping with emotional barriers and openly shared during the group discussions. Feelings of depression, social isolation, and frustration were frequently expressed in narratives. One woman compared herself to a single tree between two large buildings visible from her window; to her, the picture she took represented feeling trapped between her current disabled state and where she wanted to be (Figure 2).

For some, avoidance of these emotional barriers delayed the coping process. An African American woman initially hid the extent of her disability from others, and internalized her struggle:

“… I was helping a lot of people…because of their personality I didn’t want them to know I was sick. So I refused talking to them at all, because in my mind I felt that it was better that they be angry with me than to feel that I was sick.”

In contrast, those who described successfully coping with new physical and emotional challenges identified family, friends, and even pets as key facilitators of this process through pictures.

#### 3.2.3. Reflection: Integration versus Isolation

**Figure 3:**
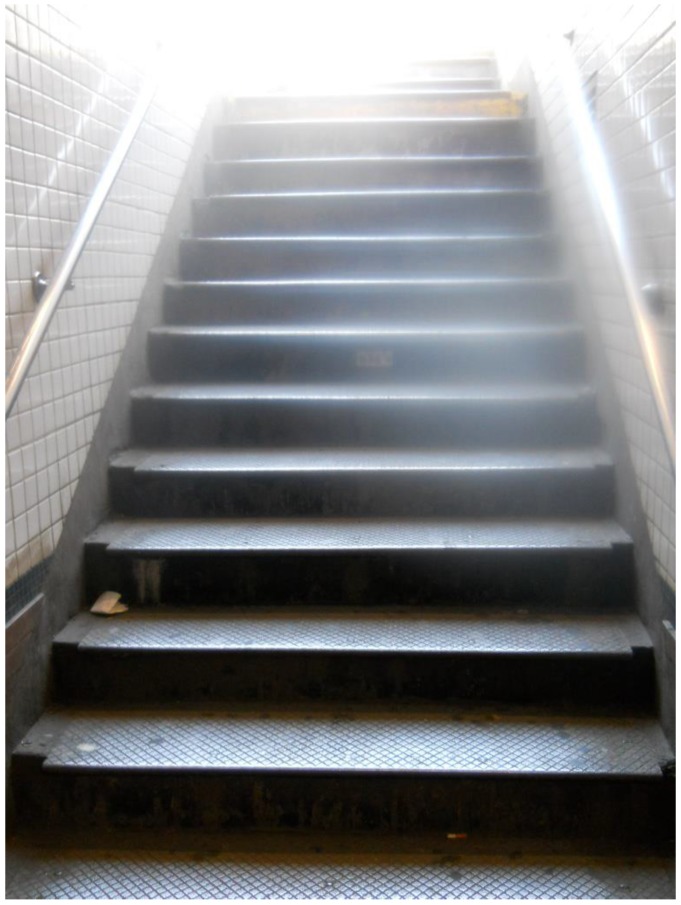
Stairs.

After the initial coping stage, participants reflected on their pre- and post-stroke experiences and described milestones by which they measured their own progress in recovery. To convey the difficulty of learning to walk again, one 46-year-old African American woman photographed a wheelchair, “to remind me of how far I came. I used to depend on that wheelchair so much.” Similarly, a 62-year-old African American man photographed subway stairs, explaining that climbing them “was one of the first things I wanted to overcome, and it’s something that I chose to overcome and I did it.” (Figure 3)

Positive reflection allowed many participants to re-evaluate their experiences as part of a coherent, purposeful journey. This perspective allowed some participants to accept their newfound limitations and adapt to new routines. After acknowledging her physical limitations, one survivor explained,

“You know, I’m not going to be the same person and I don’t really care… I’m still who I am, I’m just a little bit awkward now… Sometimes you got to go through bad…in order [to] really appreciate who you are.”

Aided by religion and/or spirituality, other participants integrated their negative experiences and limitations into a larger narrative of personal growth.

Many participants identified an increase in social activity and bonds within families as a facilitator in the reflective process. One African-American female described a sense of self-worth and empowerment from giving back to the community through the distribution of homemade juices at a local gym. Another participant explained, “I don’t think that I’m different, but…my difference is gonna make a bigger impact on somebody else. I’m a walking business card, ‘hello, your cholesterol!’”

**Figure 4:**
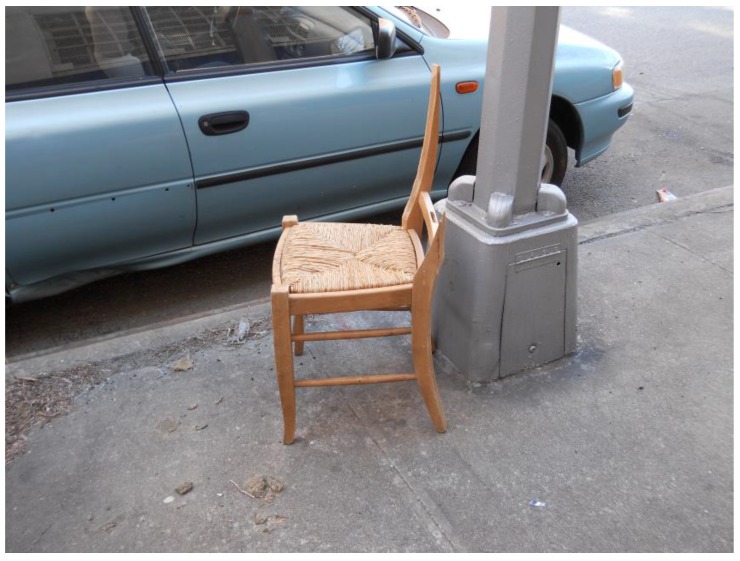
The broken chair.

However, several participants detailed a process of negative reflection accompanied by self-isolation and frustration, spending large amounts of time alone. One Hispanic female lived alone and described the negative emotions resulting from having outlived all her friends and family and shared feelings of helplessness and loss. Another participant shared a picture of a broken chair on the sidewalk and compared it to being a stroke survivor without any support (Figure 4).

“I see this broken chair and I think about how many people in my position, who had a stroke or have an illness are broken, pushed aside, discarded… if I didn’t have my family and my friends, I could have been sent to a nursing home somewhere and sit in a corner and nobody ever comes to visit, and that’s the most disheartening thing in the world.”

#### 3.2.4. Adaptation: Long-Term Changes

Ultimately, a change in perspective facilitated by positive reflection allowed many survivors to adapt to a physically and emotionally challenging environment. This allowed them to develop positive habits and lifestyle improvements. This was most often addressed during group discussions when answering “What can We Do about it?”, while discussing pictures that represented challenges faced.

• Adapting to disability

Almost all participants shared pictures of assistive devices, such as wheelchairs, shower chairs, and canes, to emphasize how a once-easy task became very difficult after the stroke. Participants framed transitions between devices (for example, from a wheelchair to a cane) as milestones and symbols of progress. 

Each group emphasized the importance of learning to do “basic” self-care activities to reestablish a sense of independence. One participant recalled, “If you want to take a shower or go outside, you don’t have to wait for anyone else to do it.” Participants described adapting to new modes of transportation. Discussing a photograph of subway stairs, one participant recalled, “I think now, I could do it by myself, but I’m nervous because there are so many stairs and you have to walk so much… I used to take the subway everywhere and now I can’t as much, now I rely on buses.”

• Diet and health awareness

**Figure 5:**
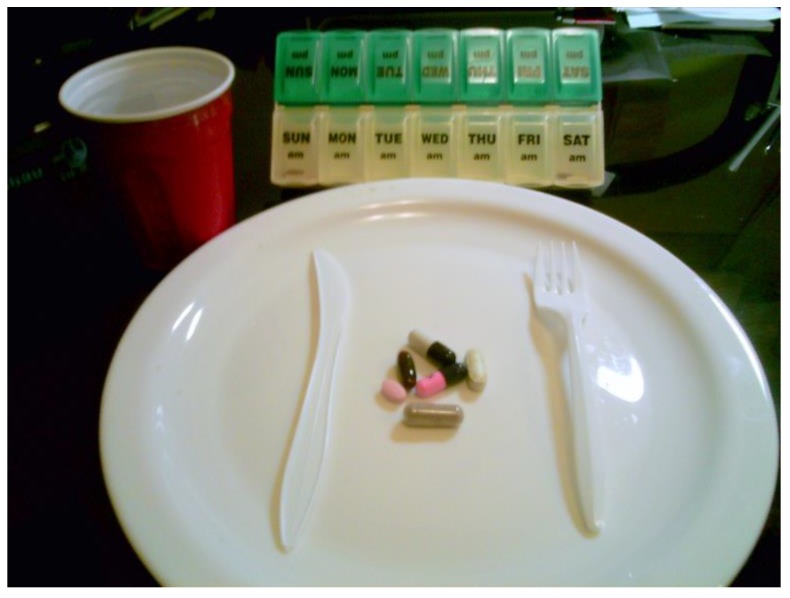
Plate of pills.

Several participants described new focus on adhering to a daily medication which some initially resisted, but ultimately adapted. Describing a picture of a plate filled with pills, one 57-year-old African American man stated,

“[The] negative is that, that’s it for life…we got to take that forever now; we don’t give it up just cause we feel better…the pills are our life… It is a positive that you’re still alive…you are living.” (Figure 5)

**Figure 6:**
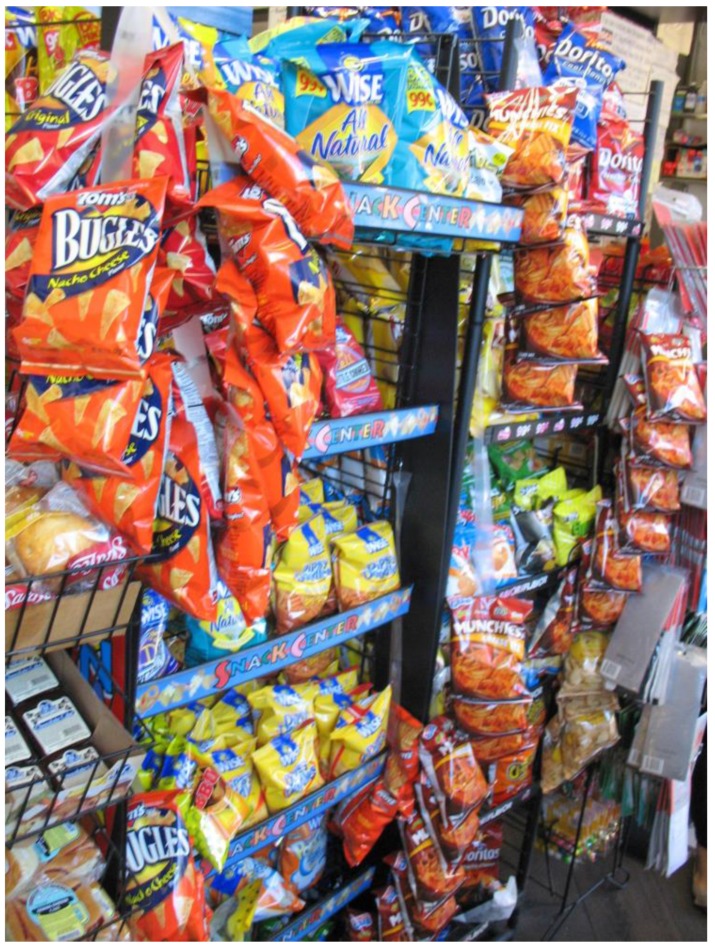
The wall of salt.

In addition, some participants identified difficulties with making healthy food choices in their neighborhood. One 62-year-old African-American male photographed snacks at a corner store, “…As soon as you walk in…they have this experience of salt…a wall full of salt…[but] with the knowledge that it will hurt you, that overcomes the desire to have it.” (Figure 6)

• Hobbies

Hobbies served as sources of motivation and physical rehabilitation; one Hispanic female became an urban gardener:

“If you had issues with yourself or with not accomplishing, you can get into gardening and forget all the negativity… When I garden I talk to myself…it’s almost like you can look at yourself and see that you’re coming along, like each day you get better.”

Aside from facilitating physical recovery, gardening and other hobbies, such as knitting, also provided survivors with opportunities to reflect on their own physical and emotional progress. In this way, hobby-based adaptation facilitated continued reflection.

#### 3.2.5. Follow-Up

• Photovoice exhibit

The results of the Photovoice sessions culminated in a gallery show highlighting pictures and allowing participants to discuss their photographs and experiences at various community venues including a premier and presentation at a legislative breakfast including ten local and state elected officials and representatives. In response to the display, officials agreed to have the local community board take up the issues highlighted such as traffic light timing, road repairs, and increasing benches on commonly used streets so people could rest along their routes. They also invited the community to display future Photovoice exhibits in the state office building, and they did, eventually displaying projects in regards to women with gestational diabetes, and people with diabetes and visual impairment. 

• Follow-up interview

All participants expressed a therapeutic value to the act of taking photographs and sharing them in a group setting. For these participants, taking part in the Photovoice project itself helped facilitate their emotional recovery. Hearing other survivors’ stories allowed them to feel less isolated in their experience. The social interactions required of group discussions gave them confidence to participate more in their communities and make new connections. One elderly African-American participant, who initially spoke of feeling very alone, became more involved in local community theater after completing the study. Another participant who initially expressed feelings of isolation stated that she developed a friendship with another participant in the group that lasted long after the sessions were over.

## 4. Discussion

Using the multifaceted technique of Photovoice, we investigated the effects of stroke on quality of life in an urban, predominantly minority population. Our study revealed a largely consistent model of major themes of the stroke experience, coping, and adaptation emerging as stages in recovery. While prior qualitative studies have produced similar models, this is the first to utilize Photovoice in a predominantly minority population of stroke survivors living in an urban area [2,14,15,16].

Individual participants navigated the immediate emotional and physical effects of stroke in different ways, but those who described a strong support network of friends and family were able to transition from initial stages of coping to long-term adaptation more easily. Isolation resulted from a failure to acknowledge physical and emotional barriers, resulting in extended coping mechanisms. Our findings are consistent with prior studies utilizing different techniques that identified social relationships as an important facilitator of physical and emotional recovery. One meta-analysis of 25 qualitative studies of stroke survivors identified social isolation as a major theme and found the majority of participants experienced feelings of increasing social withdrawal [16]. In a prior focus group study on quality of life after stroke, discussions focused on changes in social relationships post-stroke and frustration when there was a lack of social support [2].

Participants also identified environmental obstacles that uniquely affect stroke survivors living in an urban setting, such as sidewalk cracks and potholes and limited public transportation options that prevent independence. This is consistent with results from a prior study that also found lack of physical access was one of the most frequently documented barriers to recovery [17].

Several studies have explored the positive impact of creative therapy in marginalized populations, including war veterans with Post-Traumatic Stress Disorder and trauma victims; a previous Photovoice study on patients with aphasia highlighted the positive effects of the experience on its participants [18,19,20,21,22,23]. We found the Photovoice technique itself facilitated reflection on the stroke experience and the challenges in recovery. Given the positive impact of self-reflection that was seen through Photovoice, we advocate for its use as a beneficial intervention in the stroke recovery process.

Photovoice has capabilities for empowerment and self-advocacy above those found with traditional qualitative methods [24]. Several participants expressed a sense of purpose and reported feeling empowered to help others in different ways after participating; some reported increased community volunteering, while others used their stories as educational tools.

This technique also improved the relationship between the researchers and the community by allowing active engagement of the participants in the process; therefore, making the study a collaborative effort. The method was utilized in a partnership with a community action board that was focused more on action than process. For the PRAISE trial, board members reviewed every step of the process and provided input, and worked with researchers to develop the peer-education groups which were the essence of the intervention. The Photovoice intervention itself helped build a community and reduce isolation by bringing together stroke survivors from a given community. Recognizing the need to help people access positive reflection, increase social interaction, and reduce negative reflection and isolation, we changed the planned intervention in the PRAISE trial. To address isolation, we developed an introductory exercise in which we had people break into pairs, share a time when they accomplished something and share each other’s stories with the rest of the group, and we included simple exercises the group practiced regularly (i.e., ones that could be done with limited mobility or in wheelchairs) [7]. We also decided to explore whether stroke survivors were experiencing post-traumatic stress disorder (PTSD) and added specific PTSD scales to the surveys in the PRAISE trial; we were the first to find an association between stroke and PTSD and are currently developing interventions to address this [25,26].

## 5. Conclusions

Photovoice helped to identify areas in which urban, minority stroke survivors could benefit from targeted interventions. Examples include increasing outlets for social support, improving accessibility to public transportation, and providing healthier food choices in urban areas. With our participants, the active engagement fostered social interactions and facilitated empowerment that allowed participants to advocate for changes to barriers that stroke survivors face in an urban setting. In addition, the technique allowed for collaboration between researchers and participants that furthered the overall goals of the study and allowed for innovation of additional research questions. Photovoice is useful as both a tool for research and for self-reflection. Upon completion of the Photovoice study, participants expressed a sense of accomplishment and desire to share their experiences to a wider audience. This echoes the importance of reflection and socialization in transforming a potentially isolating experience into one of growth and self-realization; it illuminates the unique positive attributes this method provides in its ability to enlighten researchers and participants at the same time.

## Figures and Tables

**Table 1:** Photovoice participant demographics (n = 17)

Mean (SD)	
Age, years	64 (10)
Time since last stroke or TIA, years	2.0 (1.5)
Number (%)	
Female	11 (65)
Race/Ethnicity	
Black/African American	11 (65)
Hispanic/Latino	2 (12)
White or Other	4 (23)
Income (≤15,000)	7 (41)
Modified Rankin Score	
0–2	10 (59)
3–4	7 (41)

TIA—Transient Ischemic Attack.
